# Insights into antitrypanosomal drug mode-of-action from cytology-based profiling

**DOI:** 10.1371/journal.pntd.0006980

**Published:** 2018-11-26

**Authors:** James A. Thomas, Nicola Baker, Sebastian Hutchinson, Caia Dominicus, Anna Trenaman, Lucy Glover, Sam Alsford, David Horn

**Affiliations:** 1 London School of Hygiene & Tropical Medicine, Keppel Street, London, United Kingdom; 2 The Centre for Immunology and Infection, University of York, Heslington, York, United Kingdom; 3 Institut Pasteur, Paris, France; 4 The Francis Crick Institute, London, United Kingdom; 5 Wellcome Trust Centre for Anti-Infectives Research, School of Life Sciences, University of Dundee, Dundee, United Kingdom; Bernhard Nocht Institute for Tropical Medicine, Hamburg, Germany, GERMANY

## Abstract

Chemotherapy continues to have a major impact on reducing the burden of disease caused by trypanosomatids. Unfortunately though, the mode-of-action (MoA) of antitrypanosomal drugs typically remains unclear or only partially characterised. This is the case for four of five current drugs used to treat Human African Trypanosomiasis (HAT); eflornithine is a specific inhibitor of ornithine decarboxylase. Here, we used a panel of *T*. *brucei* cellular assays to probe the MoA of the current HAT drugs. The assays included DNA-staining followed by microscopy and quantitative image analysis, or flow cytometry; terminal dUTP nick end labelling to monitor mitochondrial (kinetoplast) DNA replication; antibody-based detection of sites of nuclear DNA damage; and fluorescent dye-staining of mitochondria or lysosomes. We found that melarsoprol inhibited mitosis; nifurtimox reduced mitochondrial protein abundance; pentamidine triggered progressive loss of kinetoplast DNA and disruption of mitochondrial membrane potential; and suramin inhibited cytokinesis. Thus, current antitrypanosomal drugs perturb distinct and specific cellular compartments, structures or cell cycle phases. Further exploiting the findings, we show that putative mitogen-activated protein-kinases contribute to the melarsoprol-induced mitotic defect, reminiscent of the mitotic arrest associated signalling cascade triggered by arsenicals in mammalian cells, used to treat leukaemia. Thus, cytology-based profiling can rapidly yield novel insight into antitrypanosomal drug MoA.

## Author summary

African trypanosomes cause devastating and lethal diseases in humans and livestock. These parasites are transmitted among mammals by tsetse flies and circulate and grow in blood and tissue fluids. There are several drugs available to treat patients but, despite their use for many decades, we know relatively little about how they work. We reasoned that exposure of trypanosomes to each drug, followed by microscopic examination of cellular structures, would reveal the major cellular compartments, structures or growth phases affected. For example, we examined two major DNA structures, and cellular compartments known as mitochondria. We found that two drugs thought to act in mitochondria did indeed disrupt this compartment, but in completely different ways. Another drug stopped cell growth at a specific point in the cycle. An arsenic-based drug, related to anti-leukaemia drugs, perturbed the nuclear DNA division cycle, indicating that arsenicals may kill parasites and cancer cells by similar mechanisms. Thus, the ‘chemical-biology’ profiles we observe illuminate distinct killing mechanisms. A similar approach can now be used to assess new drugs, and the insights may help to develop improved anti-parasite therapies.

## Introduction

Chemotherapy is central to the control of the neglected tropical diseases caused by African trypanosomes (*Trypanosoma brucei spp*), South American trypanosomes (*Trypanosoma cruzi*) and *Leishmania spp*; the related kinetoplastid parasites [1]. The current drugs suffer problems with complex administration, efficacy, toxicity and resistance [2]. There are a small number of drugs in clinical trials for these diseases but there remains a desperate need for new and improved drugs. An understanding of drug mode-of-action (MoA) would aid the development of these new drugs, but our knowledge of how the current antitrypanosomals work is lacking [1]. This has also been the case for drugs currently in clinical trials and for other promising compounds that emerged from phenotypic cell-based screening campaigns. These gaps in knowledge will only become more acute given the current trend of phenotypic screening and the typically high attrition rate during the development of compounds that emerge from target-based screening [1].

In the case of Human African Trypanosomiasis (HAT), there are five current drugs, and two in clinical trials [1]. The disease progresses from stage 1 to stage 2, when parasites invade the central nervous system, which is typically lethal without treatment. The current drugs are eflornithine, melarsoprol, nifurtimox, pentamidine and suramin. Nifurtimox and eflornithine were introduced most recently and are used in combination to treat stage 2 disease in West Africa [3]; eflornithine, however, is particularly challenging to administer. Melarsoprol use has been largely phased out, as it is highly toxic [4]. Resistance to melarsoprol, due to the disruption of a parasite aquaglyceroporin [5,6,7], is also now widespread, but this drug remains the only available treatment for stage 2 disease in East Africa; the parasite sub-species found in this region is not susceptible to eflornithine [8]. Pentamidine and suramin are used to treat stage 1 disease in West and East Africa, respectively. The new orally active drugs in clinical trials are acoziborole / SCYX-7158 [9] and fexinidazole [10].

With the exception of eflornithine and acoziborole, the mechanisms of action for the antitrypanosomal drugs above are not understood in any detail. Eflornithine enters trypanosomes via the amino-acid transporter AAT6 [11] and inhibits ornithine decarboxylase [12] and, only recently, acoziborole was shown to target an mRNA maturation factor known as CPSF3 [13]. Almost all antitrypanosomal drugs emerged based on an ability to selectively target trypanosomes or to reduce parasite viability in cell culture or in animal models. Rather than differences between host and parasite intracellular targets, the selective efficacy of suramin, melarsoprol and pentamidine is at least partly due to trypanosome-specific uptake mechanisms [14]. Suramin enters trypanosomes via an endocytic pathway involving a bloodstream stage-specific invariant surface glycoprotein ISG75 [14], and variant surface glycoprotein [15]. The action of suramin is enhanced by the import of ornithine, via an amino acid transporter [16], and its metabolism by ornithine decarboxylase, such that eflornithine is antagonistic [14]. Suramin inhibits pyruvate kinase *in vitro* but the drug may also occupy ADP/ATP binding sites in other enzymes [17], none of which have been validated as targets *in vivo*. Melarsoprol is an arsenical drug that enters trypanosomes via an adenosine transporter [18] and an aquaglyceroporin, AQP2 [5], acting primarily by forming a stable adduct, known as Mel T, with the antioxidant, trypanothione [19]. Pentamidine, like melarsoprol, enters trypanosomes via AQP2 [5]. Indeed, pentamidine inhibits the glycerol permeability of AQP2 [20] but this particular activity has little impact on parasite viability. Rather, this DNA-binding drug [21] becomes highly concentrated in the cell and collapses trypanosome mitochondrial membrane potential [22]. Pentamidine remains a low nanomolar antitrypanosomal agent against parasites lacking mitochondrial (kinetoplast) DNA, however, which display only 2.5-fold resistance [23,24]. Nifurtimox and fexinidazole are both nitro pro-drugs that are activated by a putative ubiquinone nitroreductase (NTR) located in parasite mitochondria [14,25,26], but it is unknown whether these drugs kill parasites primarily by disrupting mitochondrial functions or whether the toxic metabolites access targets outside the mitochondria.

Cytology-based profiling can facilitate antibiotic discovery efforts [27] and a selection of cellular assays have been previously applied to antitrypanosomal compounds [28]; but this previous study examined only one of the current HAT compounds (pentamidine) and employed only two of the assays described below. We now report cytology-based profiling for *T*. *brucei* to probe the MoA of all five antitrypanosomals used in patients. We describe a panel of assays that assess cell cycle progression, nuclear and mitochondrial DNA content, mitochondrial DNA replication, nuclear DNA damage, mitochondrial membrane potential, and lysosome structure and function. Using these assays, we show that each drug tested induces specific and distinct cellular perturbations, yielding novel insight into the MoA of the antitrypanosomal agents. Follow-up studies revealed a melarsoprol-induced mitotic defect that is dependent upon a specific set of kinases.

## Results

### *T*. *brucei* growth profiles during exposure to antitrypanosomal drugs

The potency of the antitrypanosomal drugs used in the clinic varies widely. The 50% effective growth inhibitory concentration (EC_50_) determined against bloodstream-form *T*. *brucei* in culture is in the low nM range for pentamidine (2.5 nM), suramin (27 nM) and melarsoprol (7 nM) but is in the low μM range for eflornithine (15 μM) and nifurtimox (2.6 μM); a 6,000-fold differential between the most potent (pentamidine) and least potent (eflornithine). It is important to note that, since EC_50_ values are typically determined over three to four days, they may fail to reflect the rate at which growth is inhibited or whether the compound is cytocidal or cytostatic at a particular concentration.

**Fig 1:**
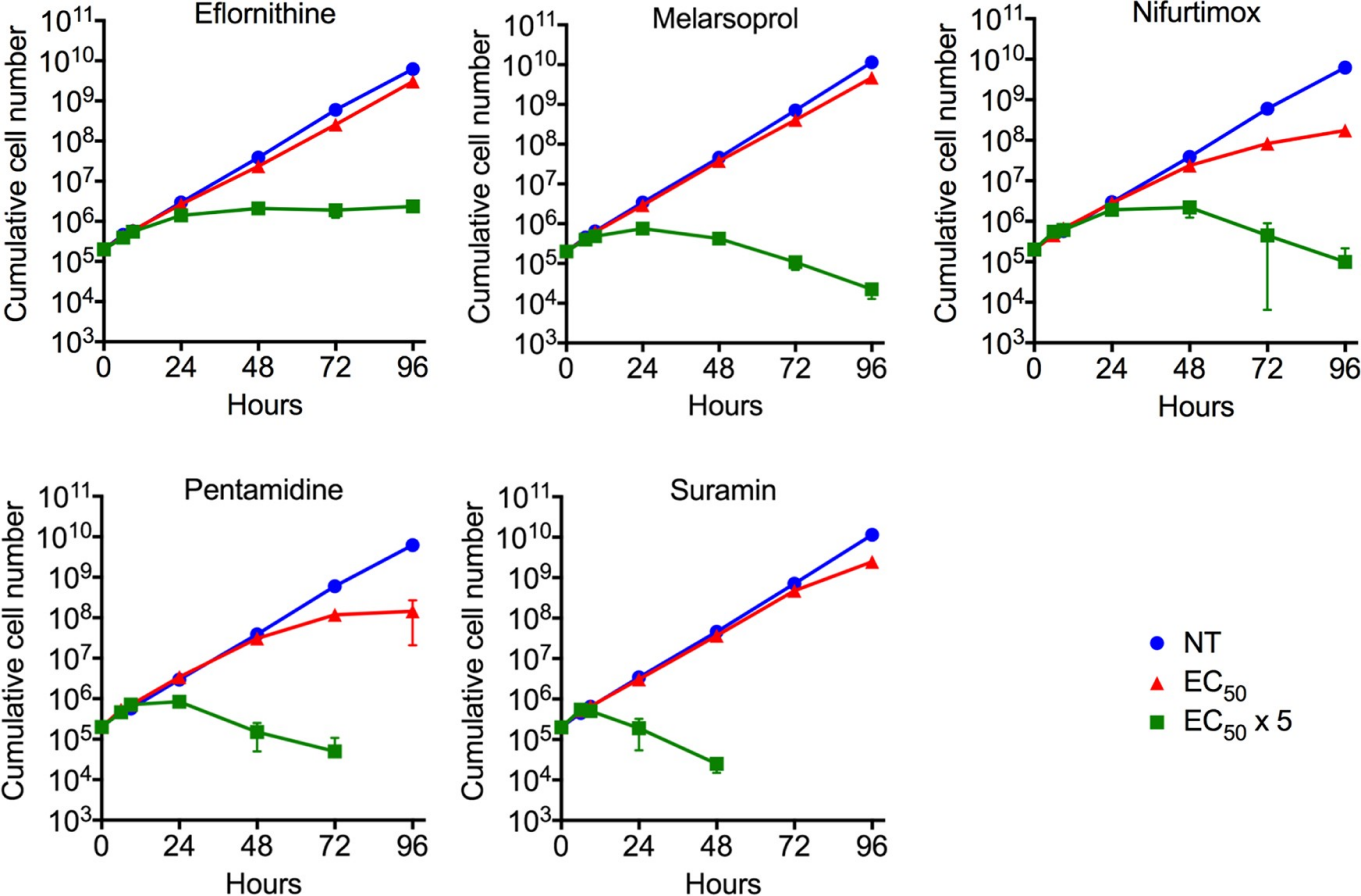
*T*. *brucei* growth profiles during exposure to antitrypanosomal drugs. Parasites were treated with each of the five clinical antitrypanosomals at 1 x EC_50_ and 5 x EC_50_ concentration. Cells that are not treated (NT) are shown as controls. Data are averages of four technical replicates. Error bars show standard deviation.

We examined the growth profiles of bloodstream-form trypanosomes treated with each of the five clinical antitrypanosomal drugs at 1 x EC_50_ and 5 x EC_50_; see Materials and methods. All drugs had a relatively moderate impact on growth at 1 x EC_50_, as expected (Fig 1). In contrast, growth at 5 x EC_50_ revealed a clear difference between eflornithine, which is known to be cytostatic [29], and the other drugs, which were all demonstrably cytocidal over four days (Fig 1). We selected 5 x EC_50_ exposure for 24 hours for subsequent assays. These concentrations and this time-point were selected to achieve a balance between allowing robust primary phenotypes to develop but to minimise the emergence of secondary effects associated with loss of viability.

Our first cellular assay was a simple examination of each of the five populations of drug-treated cells for defects in gross cellular morphology by phase-contrast microscopy. We found that the majority of suramin-treated cells became enlarged and distorted (see below). We also looked for the ‘BigEye’ phenotype, which is associated with an endocytosis-defect and observed following treatment with the *N*-myristoyltransferase inhibitor DDD85646 [30], however this phenotype was not seen.

### Melarsoprol inhibits mitosis and suramin inhibits cytokinesis

**Fig 2:**
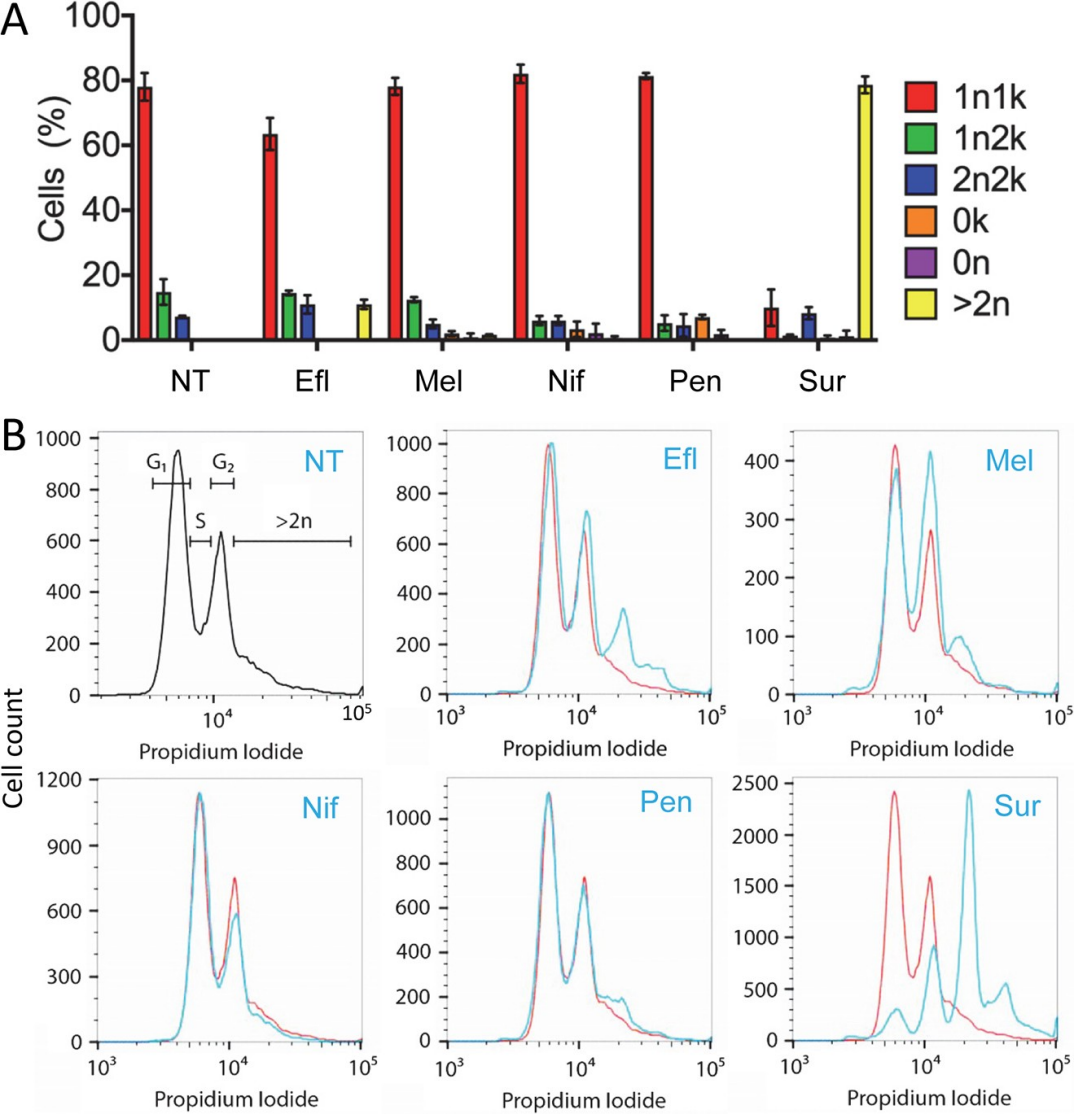
Cell cycle distribution of trypanosomes following drug treatment. (**A**) Cell cycle distribution was determined by DAPI staining and microscopy. NT, not treated; Efl, eflornithine; Mel, melarsoprol; Nif, nifurtimox; Pen, pentamidine; Sur, suramin. All drug treatments were at 5 x EC_50_ for 24 h. ‘n’ and ‘k’ refer to the number of nuclei and kinetoplasts in each cell; error bars, SD; N = 2. (**B**) Cell cycle distribution was determined by flow cytometry. Top left panel shows the gates used to calculate cell cycle stage. Other details as in A.

Arguably the simplest and most widely used fluorescent-staining method for *T*. *brucei* is DAPI-staining of DNA followed by microscopy. This is particularly informative for *T*. *brucei* because cells in which the single mitochondrial genome, or kinetoplast, has segregated are easily visualised and scored, alongside the unsegregated or segregated nuclear genome [31]. We, therefore, examined DAPI-stained cells by microscopy following drug-exposure. Cells were scored according to the number of nuclei (n) and kinetoplasts (k). In non-treated cells (Fig 2A, NT), we found that ~80% of cells displayed a 1n1k pattern (primarily G_1_ + S-phase), ~15% of cells were 1n2k (primarily G_2_) and ~5% of cells were 2n2k (post-mitosis). We exercised some caution when analysing these data for drug-treated cells, and focused on only robust phenotypes that preceded or were coincident with loss-of-viability. The analysis revealed a major increase in the proportion of cells with more than two nuclei following suramin treatment (79%), while eflornithine treatment also yielded an appreciable increase, with 11% of cells having more than two nuclei (Fig 2A, yellow bars). In addition, we noted ~5% of cells lacking a kinetoplast (0k) following pentamidine treatment (Fig 2A, orange bar, see below). This loss of kinetoplast DNA is consistent with previous observations [28].

DNA staining followed by flow cytometry allows the rapid analysis of large numbers of cells and can reveal relative cellular (primarily nuclear) DNA-content. Accordingly, we next examined propidium iodide stained cells by flow cytometry following drug-exposure. Untreated cells showed the characteristic profile, with a G_2_/M population that was approximately half the magnitude of the G_1_ population (Fig 2B, top-left and red profiles). In this assay, both nifurtimox-treated and pentamidine-treated cells conformed to the profile observed for untreated cells. In contrast, and consistent with the microscopy analysis above, we observed >70% of cells with >2n DNA content following suramin treatment; eflornithine treatment also yielded an appreciable increase in this category (Fig 2B, blue profiles). Only melarsoprol treatment yielded a second, distinct and notable perturbation using this assay; the G_2_/M population increased from 51% to 83%, relative to the G_1_-population (Fig 2B, blue profile). Since we had not scored an increase in the proportion of 2n cells by microscopy following melarsoprol-treatment, this particular flow-cytometry profile indicated an increase in cells with a replicated but unsegregated nuclear genome. Thus, DNA-staining with microscopy and flow cytometry suggested that suramin inhibited cytokinesis and that melarsoprol inhibited mitosis (see below).

### DNA replication in kinetoplasts and DNA damage in nuclei

**Fig 3:**
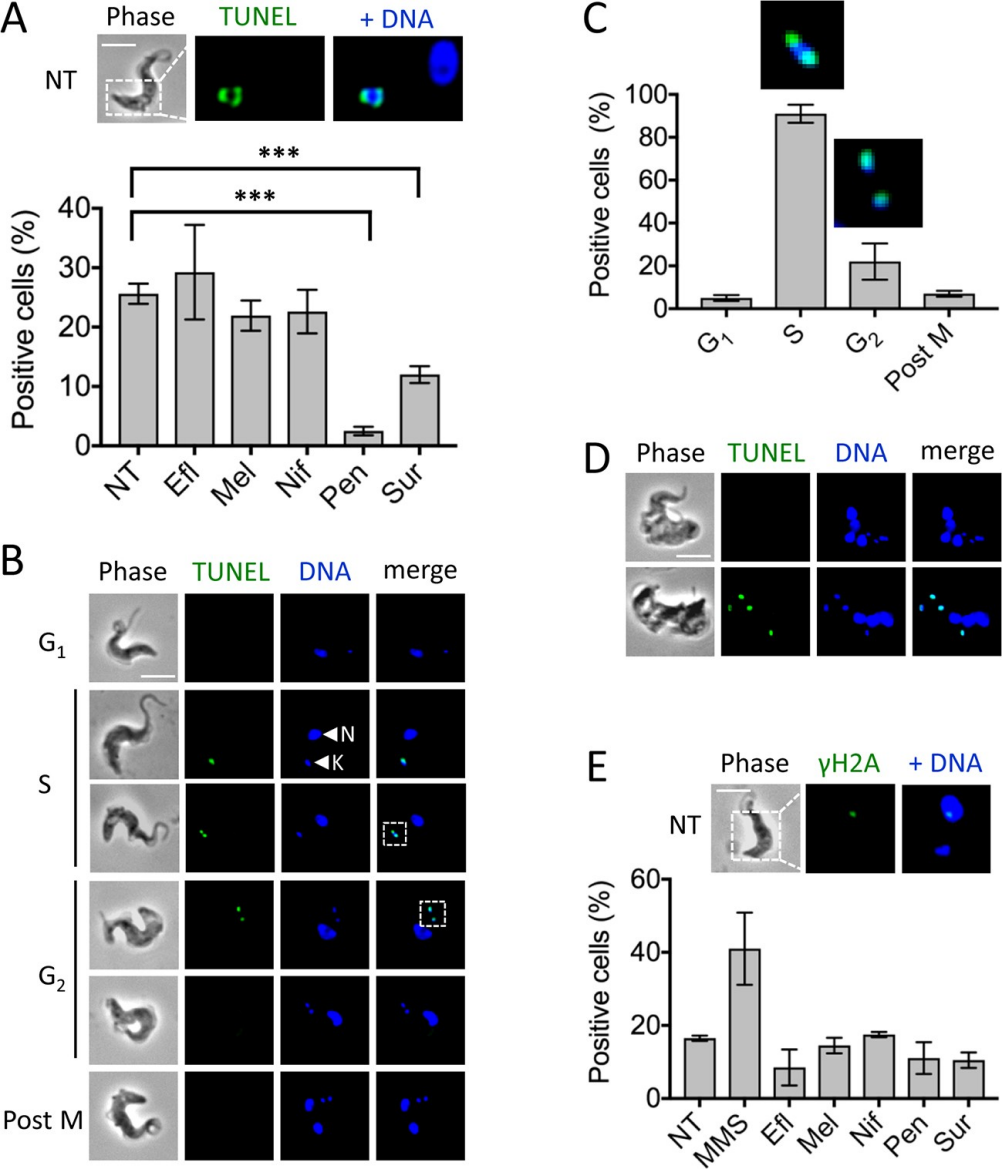
Assessment of ‘DNA-breaks’ in nuclei and gaps in kinetoplast circles. (**A**) Percentage of cells positive for a TUNEL signal. ***, *P* < 0.001 from Students *t*-test. The microscopy images show an example of the kinetoplast-associated TUNEL signal in NT cells. (**B**) Example images of TUNEL signals, always associated with the kinetoplast (k) rather than the nucleus (n), at different cell cycle stages; G_1_, S, G_2_ and post M (post mitotic). Dashed boxes indicate magnified regions shown in C. (**C**) Percentage of cells in each cycle stage positive for TUNEL signals, inset images show magnified signals from B as indicated by dashed boxes. (**D**) Examples of TUNEL signals in none or all four kinetoplasts following suramin treatment. (**E**) Percentage of cells positive for γH2A signals. The microscopy images show an example of the nuclear γH2A signal in NT cells. MMS, methyl methanesulfonate. Other details in A, C and E as in Fig 2A; error bars, SD; N = 2; all scale bars, 5 μm.

The TUNEL (*T*erminal deoxynucleotidyl transferase d*U*TP *N*ick *E*nd *L*abelling) assay allows the fluorescent labelling and detection of blunt DNA ends, following programmed cell-death (PCD) for example; notably, conventional PCD is not thought to operate in trypanosomatids [32]. Using this assay, TUNEL-signals were not readily detected in trypanosome nuclei. In contrast, and as previously reported [33], we observed robust signals associated with kinetoplasts (Fig 3A). We found approximately 25% of control cells to be TUNEL-positive (Fig 3A). Eflornithine, nifurtimox and melarsoprol-treatment did not significantly alter the proportion of cells with detectable TUNEL-signals when compared to cells that had not been exposed to drug (Fig 3A), but the proportions of TUNEL-positive cells were significantly reduced following pentamidine or suramin-treatment (Fig 3A). This was likely due to loss of kinetoplast DNA following pentamidine-treatment (see above) and may have been due to limited repeated rounds of kinetoplast DNA replication in multi-nucleated cells following suramin-treatment (see below).

TUNEL signals were found to be cell cycle dependent (Fig 3B), consistent with the high concentration of transient nicked DNA ends expected to be present during the replication of thousands of minicircles [34]. Indeed, the majority (91%) of elongated kinetoplasts (in S-phase cells) and a substantial proportion (22%) of segregated kinetoplasts (in G_2_ cells) were TUNEL-positive, while we observed very few TUNEL-positive kinetoplasts in G_1_ or post-mitotic cells (Fig 3C). The TUNEL-signals were consistently observed at opposite poles of extended kinetoplasts and, when present, on both segregated kinetoplasts. Notably, the appearance of kinetoplast-associated TUNEL-signals remained synchronised even in those suramin-treated cells with four kinetoplasts; all four were either negative or positive in each cell (Fig 3D). Thus, TUNEL revealed those cells that are progressing through kinetoplast S-phase and indicated continued synchronisation, even in cells with four kinetoplasts.

We previously identified trypanosome γH2A, a modified histone H2A that is phosphorylated at the *C*-terminus and that accumulates at sites of nuclear DNA double-strand breaks [35]. To assess nuclear DNA damage in drug-treated cells, we used a γH2A antibody in an immunofluorescence assay; methyl methanesulfonate (MMS) is a radiomimetic DNA-damaging agent and was included as a positive control (Fig 3E). We found no significant differences in the proportion of cells with nuclear γH2A foci compared to untreated cells (Fig 3E), suggesting that none of the current drugs kill trypanosomes by forming double-strand breaks in nuclear DNA. Notably, the nitro pro-drugs, nifurtimox and fexinidazole, although found to be mutagenic by Ames test (*Salmonella typhimurium* based assay), are not thought to be genotoxic to mammalian cells [36,37].

### Both nifurtimox and pentamidine disrupt mitochondrial membrane potential

**Fig 4:**
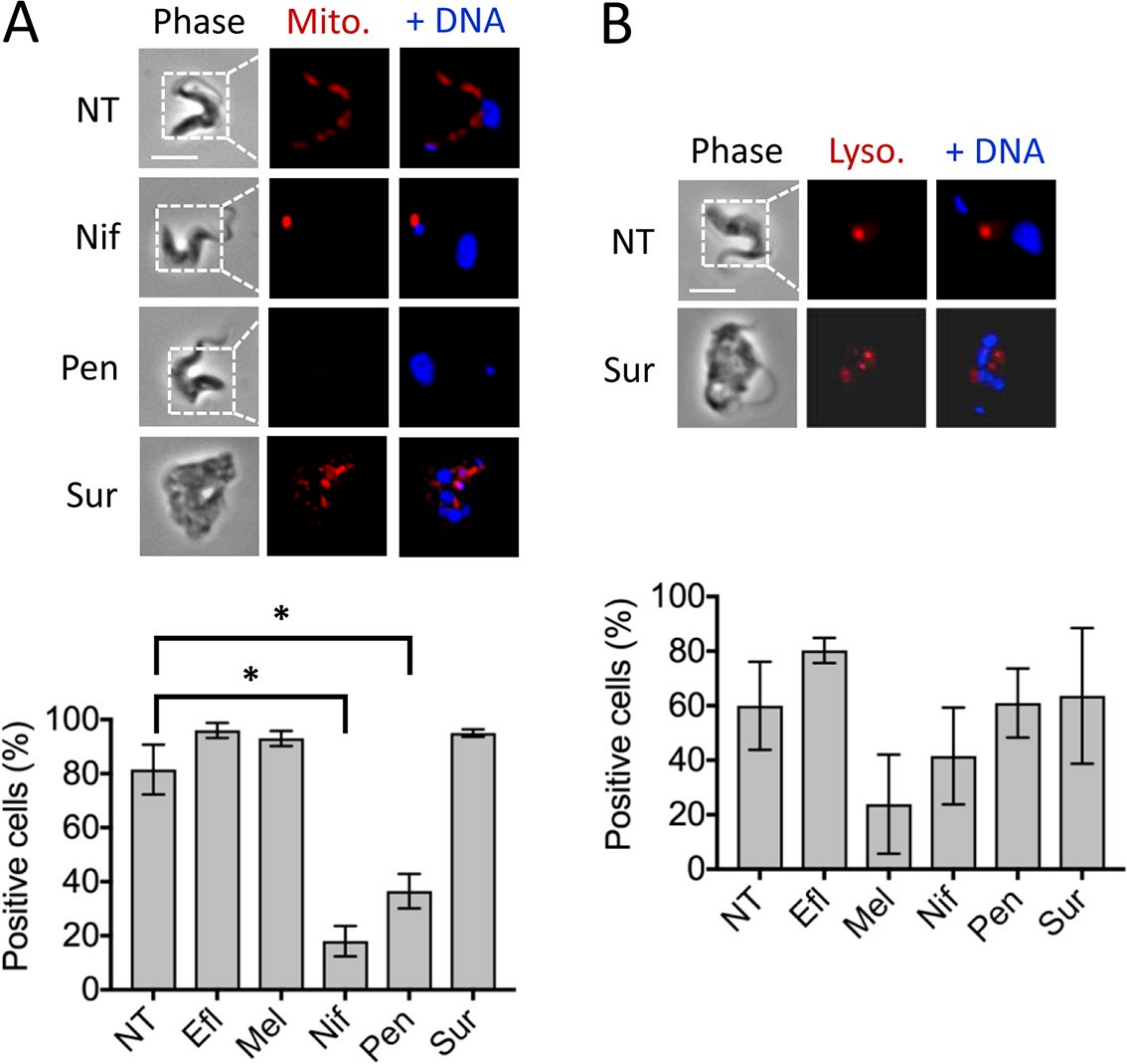
Assessment of mitochondrial and lysosomal phenotypes. (**A**) The microscopy images show examples of the Mitotracker signals. Percentage of cells positive for the typical ‘extended’ Mitotracker signal are shown. *, *P* < 0.05 from Students *t*-test. (**B**) The microscopy images show examples of the Lysotracker signals observed in untreated and suramin-treated cells. Percentage of cells positive for Lysotracker signal are shown. Other details as in Fig 2A; error bars, SD; N = 2; scale bars, 5 μm except for suramin-treated cells, which are 10 μm.

MitoTracker fluorescence staining is dependent upon mitochondrial membrane potential. We treated trypanosomes with antitrypanosomal drugs and scored cells by microscopy for an extended MitoTracker signal, as observed in non-treated cells (Fig 4A, NT panels). Significantly fewer cells scored positive for extended signals following either nifurtimox or pentamidine-treatment, compared to non-treated cells (Fig 4A). There were also qualitative differences in signals resulting from drug treatments; whereas pentamidine eliminated the detectable signal, nifurtimox-treatment produced an intense and discreet signal adjacent to the kinetoplast (Fig 4A). Notably, mitochondrial membrane potential was maintained following suramin-treatment (Fig 4A), despite the gross morphological perturbations observed in these cells.

LysoTracker is highly selective for acidic organelles. We treated trypanosomes with antitrypanosomal drugs and scored cells for LysoTracker signals by microscopy. Non-treated cells displayed a single signal between the nucleus and kinetoplast (~60%), as expected for the trypanosome lysosome (Fig 4B, NT panels). Melarsoprol substantially reduced the proportion of LysoTracker-positive cells (Fig 4B), but this failed to achieve statistical significance. In the case of suramin, which is known to accumulate in trypanosomes through receptor-mediated endocytosis [14], acidification of the lysosomal compartment does not appear to be perturbed (Fig 4B). The variability we observe in this assay suggests either that additional replicates will be desirable in future, or that this particular assay will be of value only when major lysosomal perturbation occurs.

### Pentamidine induces progressive loss of kinetoplast DNA

**Fig 5:**
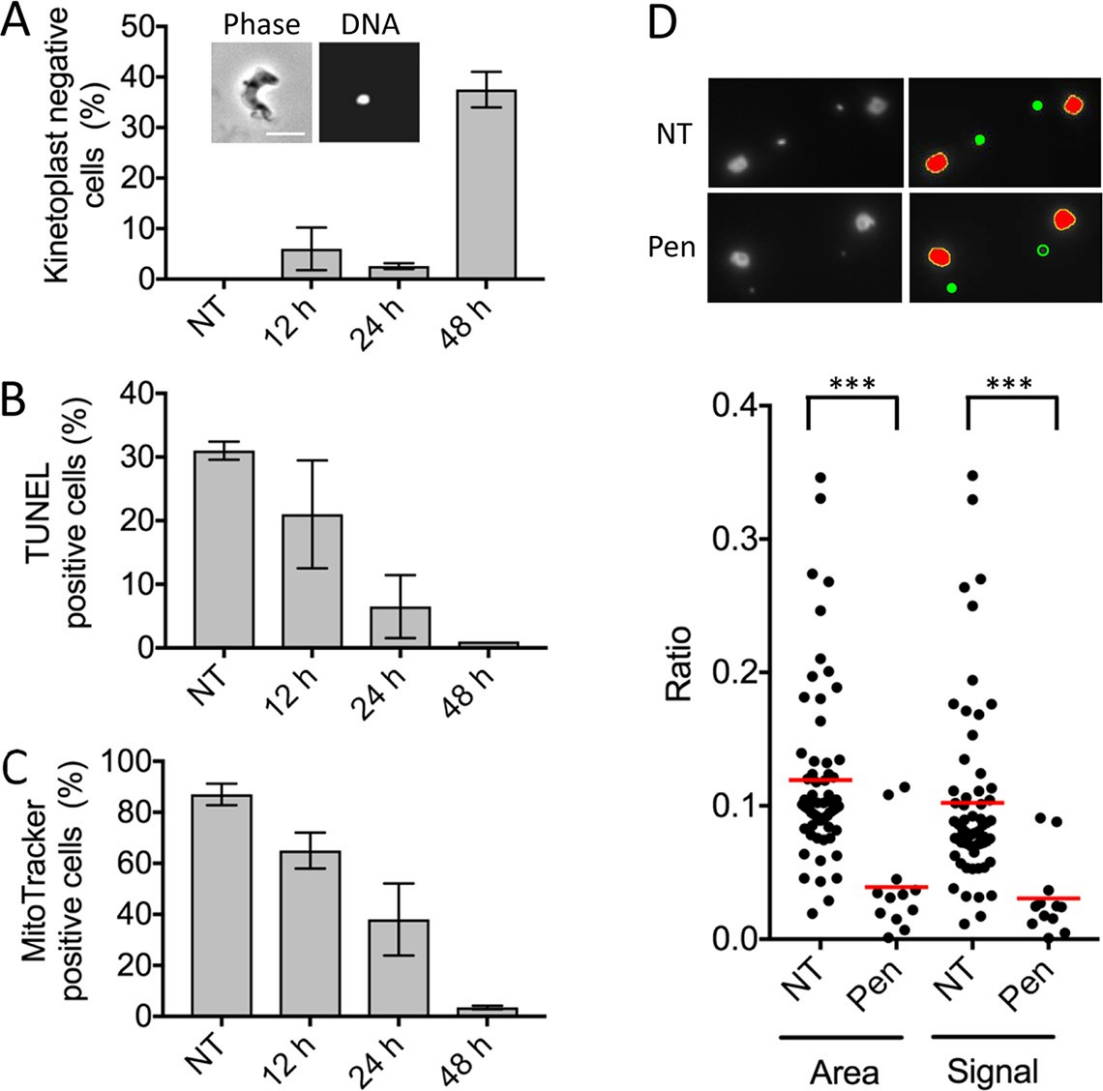
Pentamidine induces progressive loss of kinetoplast DNA. Effects of pentamidine treatment for 12, 24 and 48 h on (**A**) percentage of kinetoplast negative cells, (**B**) percentage of TUNEL-positive cells and (**C**) percentage of extended Mitotracker signal positive cells. The inset in A shows an example of a cell with no detectable kinetoplast DNA; error bars, SD; N = 2; scale bar, 5μm. (**D**) ImageJ analysis. Raw images (left hand panels) and ImageJ outputs (right hand panels) demonstrating that kinetoplasts fall below the threshold of detection (open green circle). Ratios of kinetoplasts to nuclei, total area and total signal (area multiplied by mean intensity) are plotted for cells with detectable kinetoplasts. Red lines, averages; ***, *P* < 0.001 from Students *t*-test.

Using the DAPI, TUNEL and MitoTracker assays following pentamidine-treatment, we observed kinetoplast-loss, a reduced proportion of positive cells and a loss of mitochondrial membrane potential, respectively. To quantify these effects, we scored each phenotype following 12, 24, and 48 h of drug-exposure (Fig 5A–5C). These analyses revealed a sharp increase in kinetoplast-negative cells in the 48-h DAPI-stained population (Fig 5A) and a progressive decline in TUNEL-positive (Fig 5B) and MitoTracker-positive cells (Fig 5C) at each time-point.

DAPI-stained cells appeared to reveal progressively diminished kinetoplast DNA rather than loss in one step. For a quantitative and objective assessment of kinetoplast DNA-staining intensity, we adapted an ImageJ-based approach [31]; see Materials and methods. ImageJ efficiently identified nuclei and kinetoplasts in untreated cells (Fig 5D, red and green, respectively in the upper panel), but failed to identify very low-intensity kinetoplasts in pentamidine-treated cells (Fig 5D, open green circle in the lower panel). A quantitative analysis indicated that the kinetoplasts that are still detected following pentamidine-treatment display reduced surface area and signal intensity relative to the control population; values are expressed relative to nuclei in the same cell (Fig 5D). These results confirm progressive loss of kinetoplast DNA induced by pentamidine.

### Nifurtimox induces a specific reduction in mitochondrial protein abundance

**Fig 6:**
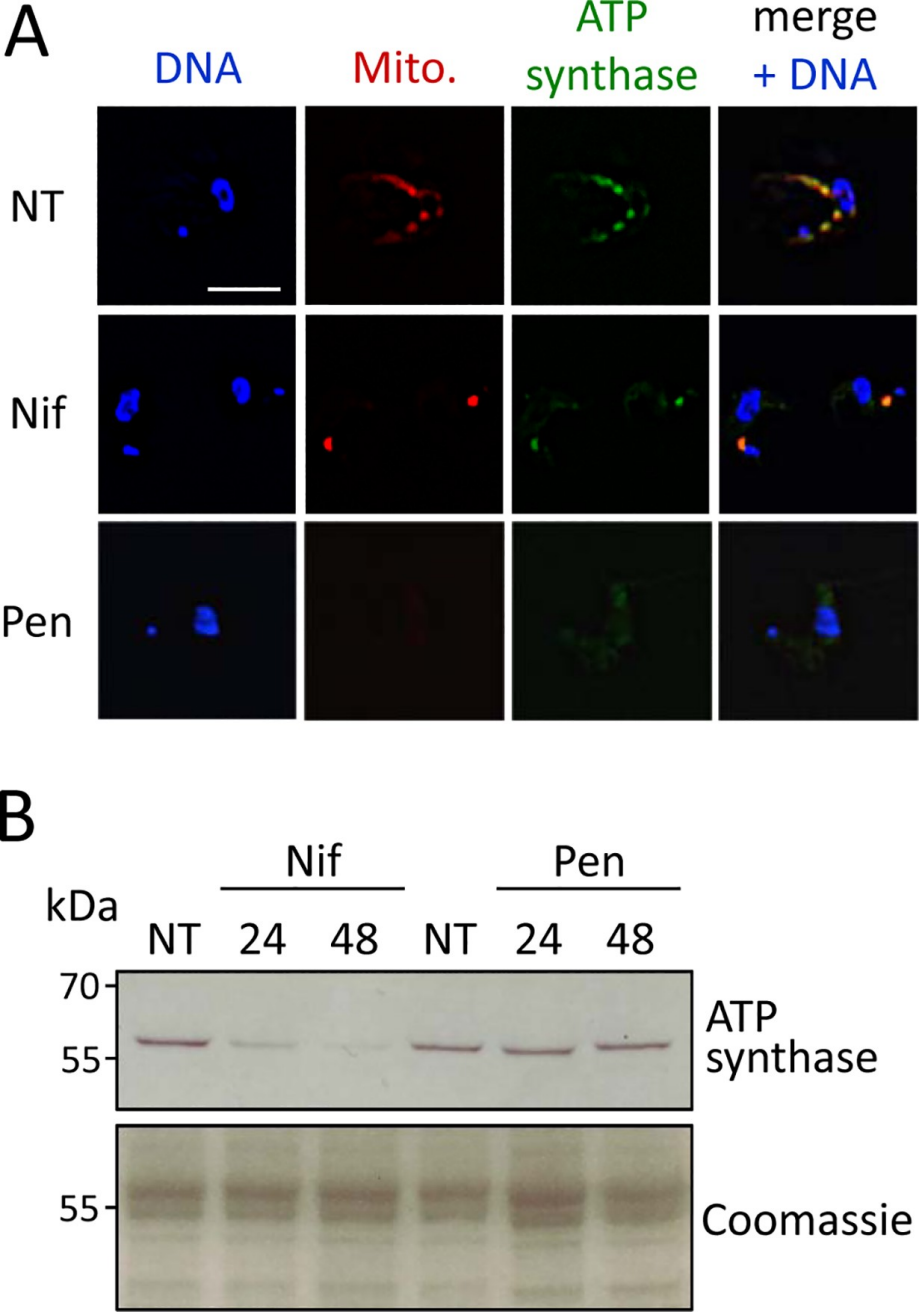
Nifurtimox induces a specific reduction in mitochondrial protein abundance. (**A**) The microscopy images show the Mitotracker signals and F1β mitochondrial ATP-synthase subunit following nifurtimox or pentamidine treatment. Scale bar, 5 μm. (**B**) Protein blotting to detect the F1β mitochondrial ATP-synthase subunit following nifurtimox or pentamidine treatment; predicted size, 55.8 kDa.

Nifurtimox-treatment produced an intense and discreet MitoTracker signal adjacent to the kinetoplast (Fig 4A). To explore this effect further, we stained the nuclear-encoded F1β-subunit of the mitochondrial ATP-synthase (Tb927.3.1380) in drug-treated and MitoTracker-stained cells and examined these cells by microscopy. The ATP-synthase signal was largely coincident with the extended MitoTracker signal in control cells (Fig 6A, NT panels) and was also coincident with the focal MitoTracker signal in nifurtimox-treated cells (Fig 6A, Nif panels), possibly indicating a structural defect in mitochondria in the latter cells. We next assessed F1β-subunit expression by protein blotting of whole-cell extracts, which revealed a substantial and specific reduction in abundance following nifurtimox-treatment (Fig 6B). Depletion of the ATP-synthase component may reflect a more widespread depletion of mitochondrial proteins, consistent with activation of this pro-drug by a mitochondrial NTR [14,25,26]. Using the same assays, pentamidine-treated cells displayed a diffuse and cytosolic, rather than mitochondrial, ATP-synthase signal (Fig 6A, Pen panels). In this case, protein import into mitochondria [38] may be defective as a result of loss of mitochondrial membrane potential, as indicated by the diminished MitoTracker signal (Fig 6A, Pen panels). Consistent with this hypothesis, the total cellular F1β signal detected by protein blotting remained constant following pentamidine treatment (Fig 6B).

### Melarsoprol inhibits mitosis by a mechanism that is suppressed by multiple kinases

**Fig 7:**
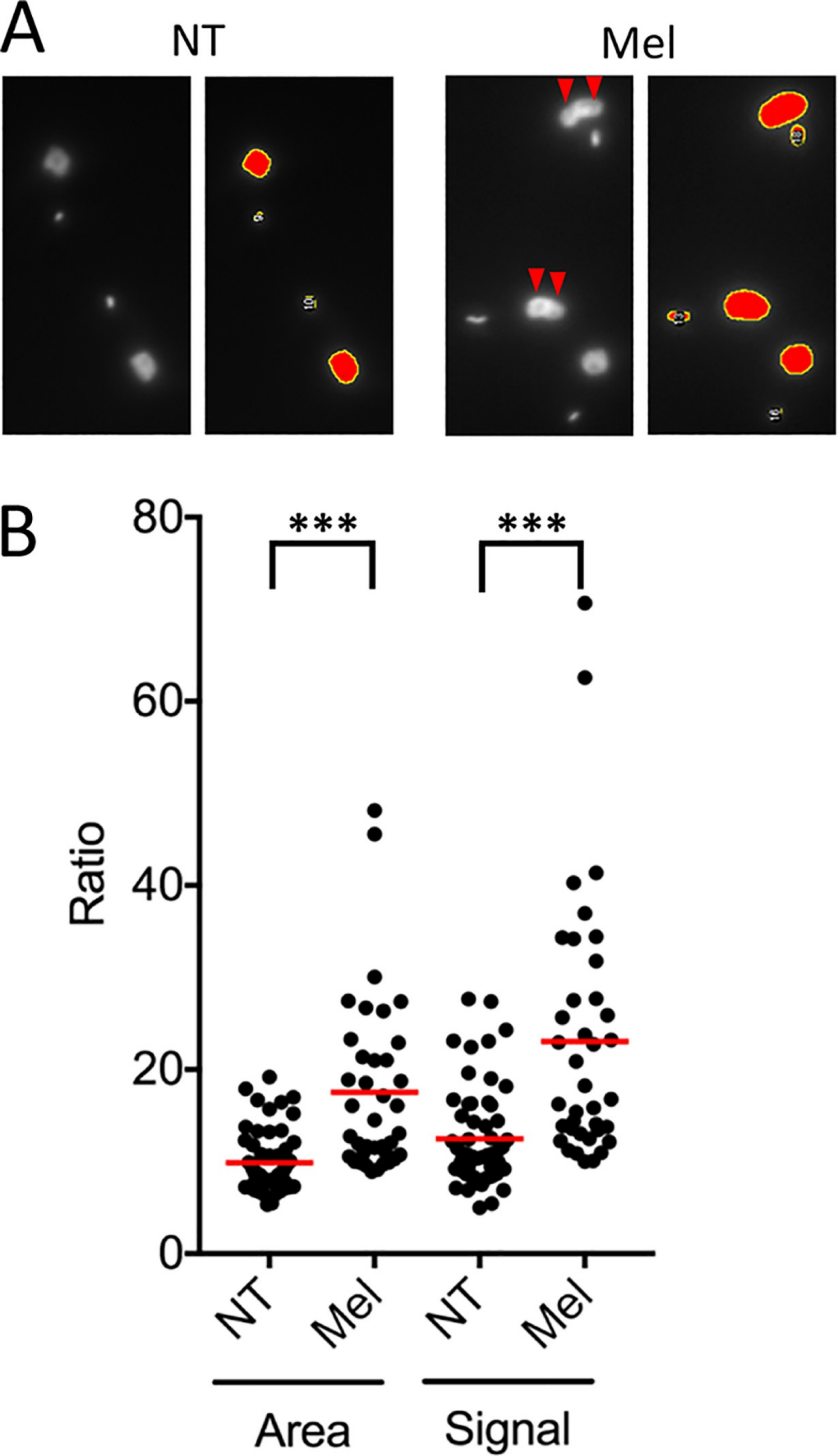
Quantitative analysis of the melarsoprol-induced mitotic defect. (**A**) ImageJ analysis. Raw images (left hand panels) and ImageJ outputs (right hand panels) demonstrating conjoined nuclei (red arrowheads) after melarsoprol treatment. (**B**) DNA content of nuclei in melarsoprol (Mel) treated and non-treated (NT) cells is plotted. Ratio of area and signal (area multiplied by average intensity) of nuclei to average area and signal of kinetoplasts in ‘1n2k’ cells. Error bars, SD; red lines, averages; ***, *P* < 0.001 from Students *t*-test.

DNA staining followed by microscopy or flow cytometry suggested that melarsoprol inhibited mitosis. Indeed, a closer inspection of DAPI-stained cells revealed a sub-set (>10%) with “conjoined” nuclei (Fig 7; top panels, double-arrowheads). For an objective assessment of nuclear DNA-staining intensity in these cells, we used the quantitative ImageJ-based approach; see Materials and methods. In this case, nuclei were compared to kinetoplasts in ‘1n2k’ cells, revealing both increased nuclear surface area and signal intensity following melarsoprol-treatment (Fig 7). These quantitative results confirmed the mitotic defect resulting from melarsoprol treatment.

**Fig 8:**
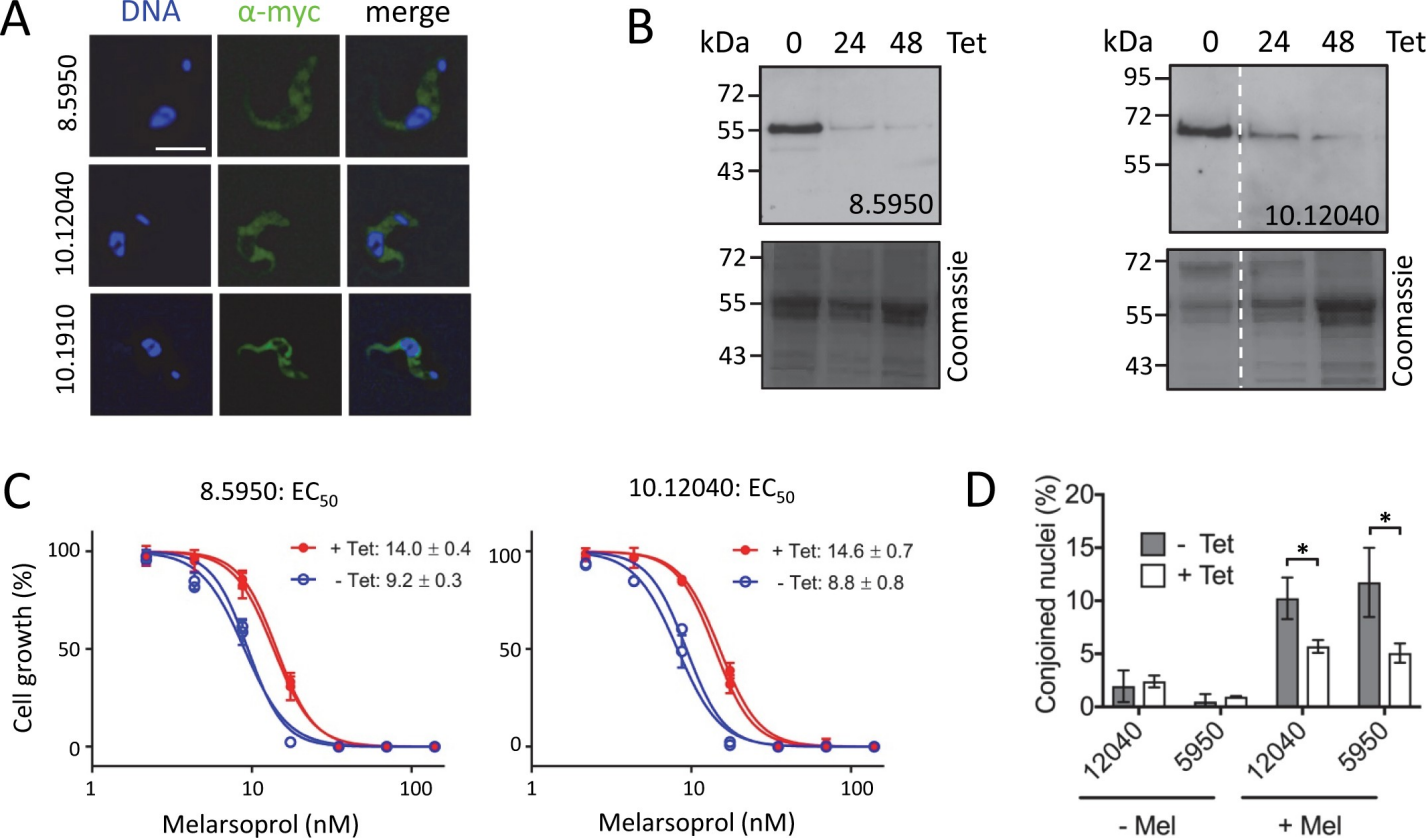
Melarsoprol inhibits mitosis by a kinase-dependent mechanism. (**A**) The microscopy images show the localisation of three *C*-terminal epitope (myc) tagged kinases. Scale bar, 5 μm. (**B**) Protein blotting indicates tetracycline (Tet)-inducible RNAi-mediated knockdown of the indicated kinases. Coomassie-stained gels serve as loading-controls. (**C**) Dose-response curves for melarsoprol following knockdown (+Tet) of the indicated kinases. EC_50_ values are shown +/- SD. Data are shown for pairs of biological replicates; similar data were also obtained for a third biological replicate in each case. (**D**) The melarsoprol-induced conjoined nuclei phenotype is diminished following knockdown of the indicated kinases. *, *P* < 0.05 from Students *t*-test.

Cytology-based profiling may be combined with orthogonal approaches to probe antitrypanosomal compound MoA. We have found high-throughput genetic screening to be particularly informative [13,14]. A prior RNA interference (RNAi) Target Sequencing (RIT-seq) screen revealed a highly significant (*P* = 2.3 × 10^−9^) over-representation of kinases, comprising three hits among the top seven in this screen; these were a putative mitogen-activated protein kinase (MAPK11, Tb927.10.12040), a putative MAPK kinase (MKK4, Tb927.8.5950) and a putative MAPK kinase kinase (STE11, Tb927.10.1910) [14]. It is also notable that trypanosome CDC14 (Tb927.11.12430) was a hit in this screen, since human Cdc14 stabilises Wee1, a key kinase that inhibits mitosis [39]. Thus, knockdowns that promote mitosis may partially counteract the inhibitory effects of melarsoprol, but the RIT-seq screening hits noted above have not previously been independently validated nor further characterised. To explore our hypothesis, we further characterised these kinases. All three were expressed with a *C*-terminal 12-myc epitope tag and the tagged proteins were all found to localise to the trypanosome cytosol (Fig 8A). Since RIT-seq screening fails to yield clonal knockdown strains [14], we assembled pairs of independent knockdown strains for two of the kinases (Tb927.10.12040 and Tb927.8.5950). Both knockdowns, confirmed by monitoring the epitope-tagged proteins (Fig 8B), reproducibly and significantly (*P* < 0.0001) increased melarsoprol EC_50_ by 1.7 +/- 0.07 fold and 1.5 +/- 0.01 fold, respectively (Fig 8C), as predicted by the RIT-seq screen [14]. Finally, we determined whether knockdown alleviated the melarsoprol-induced, conjoined-nuclei phenotype and found that this was indeed the case for both kinases (Fig 8D). Thus, the melarsoprol-induced mitotic defect is kinase-dependent.

## Discussion

Cytocidal or cytostatic compounds identified using phenotypic approaches may target proteins, nucleic acids, membranes or other metabolites. They may also exhibit polypharmacology, killing cells by perturbing multiple pathways. Even compounds developed as target-based therapies may kill trypanosomes by ‘off-target’ mechanisms. Determining mechanism of action remains a major challenge for these drugs and compounds and an improved understanding of how they kill parasites could present new opportunities in terms of developing more potent compounds, delivering compounds to their targets more effectively or devising combination therapies that minimise the likelihood of resistance. Eflornithine kills trypanosomes by inhibiting ornithine decarboxylase but the mode-of-action of the other four current antitrypanosomal drugs is not known. Our studies indicate that cytology-based profiling can provide a rapid and effective means to yield insight into drug mode-of-action and we now present additional insights into the mode-of-action for all current drugs used to treat human African trypanosomiasis.

Suramin was found to produce cells with more than two nuclei, indicating a defect in cytokinesis with continued mitosis. Multi-nucleated cells were still stained by MitoTracker, indicating that this organelle retained membrane potential. DAPI-staining and TUNEL (terminal dUTP nick end labelling) assays indicated that nuclear:nuclear and nuclear:kinetoplast replication remained synchronised in these cells. Indeed, although the TUNEL assay failed to reveal any nuclear DNA damage induced by the drugs used here, it did provide an excellent marker for the kinetoplast replication cycle, producing robust signals consistent with the presence of DNA-ends at antipodal sites on the kinetoplast during minicircle DNA replication [34]. In this regard, TUNEL provides a useful marker for kinetoplast S-phase.

Pentamidine is a DNA-binding drug [21] that collapses trypanosome mitochondrial membrane potential [22] and induces loss of kinetoplast DNA [28]. Data from *Saccharomyces cerevisiae* indicated pentamidine accumulation in the mitochondrion but also inhibition of translation in these cells [40]. In addition, metabolomic studies indicated that pentamidine is unlikely to act through the inhibition of any specific metabolic pathways [41]. We find that pentamidine-induced loss of kinetoplast DNA is progressive, suggesting progressive loss of maxi- and minicircles; the latter are present in thousands of copies per kinetoplast [34]. Kinetoplast DNA loss is revealed by both DAPI-staining and TUNEL-assay. Loss of kinetoplast DNA is expected to disrupt membrane-potential since the A6-subunit of the ATP-synthase, required to maintain this potential, is encoded by kinetoplast DNA [42]. Thus, we suggest that kinetoplast DNA is indeed a primary target for pentamidine, involving inhibition of mitochondrial type II topoisomerase action, as previously suggested [43]. What remains unclear is whether loss of mitochondrial membrane-potential is solely a consequence of the kinetoplast defect or whether this reflects an independent response to pentamidine.

Nifurtimox is a pro-drug that is activated by a mitochondrial NTR [26], but it is unknown whether the toxic metabolite(s) access(es) targets outside the mitochondrion, or whether parasite killing is primarily due to disruption of mitochondrial functions. We found that nifurtimox, like pentamidine, also disrupted the MitoTracker signal. Kinetoplast defects were not observed, however, and the MitoTracker staining pattern was distinct to that seen with pentamidine. Nifurtimox also induced loss of a nuclear-encoded ATP-synthase subunit, while data from pentamidine-treated cells indicated that ATP-synthase was still expressed, but not imported, when membrane potential was perturbed. Thus, we suggest severe disruption of mitochondrial structure and function by nifurtimox, consistent with damage to targets in the organelle where the drug is activated. Fexinidazole is another nitro pro-drug, currently in clinical trials, that is also activated by mitochondrial NTR [25]. These drugs may act through similar antitrypanosomal mechanisms.

Melarsoprol forms potentially toxic adducts with trypanothione [19], and we now show that this drug increases the proportion of cells with a replicated but unsegregated nuclear genome. This indicates a defect in mitosis. The identification of multiple putative kinases in a loss-of-function screen for melarsoprol-resistance suggested a role for a signalling cascade in melarsoprol susceptibility [14]. The current findings led us to consider a more specific role for these putative kinases in the control of mitosis. Indeed, these proteins are putative MAPK, MAPKK and MAPKKKs and our results now indicate that the mitotic defect is less pronounced when the putative MAPK (Tb927.10.12040) and MAPKK (Tb927.8.5950) are depleted. We suggest that these kinases negatively control mitosis, as part of normal quality control following DNA replication. Bypass of this surveillance could allow cells to continue to grow, possibly accumulating, but tolerating, melarsoprol-induced (oxidative) damage. Thus, knockdowns that promote mitosis may partially counteract the inhibitory effects of melarsoprol.

Arsenicals are used to treat leukemia but are also themselves mutagens. There is indeed evidence that arsenic induces DNA damage in yeast [44]. Notably, the MoA in yeast involves activation of a MAP kinase pathway [45] and this also appears to be the case in mammalian cells [46], where mitotic arrest occurs as a result of induction of a mitotic spindle checkpoint [47]. We did not detect any evidence for nuclear DNA damage in *T*. *brucei* following melarsoprol treatment but, as detailed above, did find a melarsoprol-dependent and kinase-dependent mitotic defect. Thus, arsenical MoA may operate through a common kinase signalling cascade leading to mitotic arrest in both trypanosomes and mammalian cells. Notably, like the Myt1 kinase, which phosphorylates Cdc2 and controls mitosis in *Xenopus* [48], Tb927.10.1910 has a putative transmembrane domain; Tb927.10.1910 also has a putative guanylate cyclase domain. These findings illustrate how cytology and genetic approaches can converge to yield insights into drug MoA in trypanosomes. In this case, we propose a common MoA for arsenicals in human cells and in trypanosomes.

Cytology-based approaches can provide rapid and cost-effective methods for quantitative profiling of cellular responses to drugs. The drugs can also be considered as chemical probes for exploring parasite biology. Taking a systematic cytology-based approach with antitrypanosomal drugs of uncertain MoA, our findings indicate target organelles and structures for pentamidine, nifurtimox and melarsoprol; the kinetoplast, the mitochondrion and the nucleus, respectively. Further analysis, guided by the primary assays, indicated destruction of mitochondrial ATP-synthase by nifurtimox and a mitotic defect induced by melarsoprol. These assays should also provide novel insights into MoA when applied to cells treated with other antitrypanosomal compounds, including those with known primary targets. Common profiles will allow compounds to be clustered based on their primary MoA. New assays and bespoke assays can be incorporated as appropriate and these may be guided by outputs from orthogonal genetic, proteomic, metabolomic or computational approaches. The approach may also be usefully applied to the other parasitic trypanosomatids, *T*. *cruzi* and *Leishmania*. These and other approaches should reveal those particularly susceptible pathways that can be prioritised and targeted by antitrypanosomal therapies.

## Materials and methods

### Strains and drugs

Lister 427 derived *T*. *brucei* (clone 221a) bloodstream form cells were grown in HMI-11 in the presence of antitrypanosomal drugs. Cultures were initiated at 2 x 10^5^ cells.ml^-1^ and incubated at 37°C in a humidified 5% CO_2_ atmosphere. The drugs were applied at five times the EC_50_, as determined using a standard AlamarBlue assay [49]; for EC_50_ determination, drug exposure was for 66–67 h and AlamarBlue incubation was for 5–6 h. Plates were read on an Infinite 200 Pro plate-reader (Tecan). The EC_50_ values of the antitrypanosomal drugs used were: Eflornithine 15 μM; Melarsoprol 7 nM; Nifurtimox 2.6 μM; Pentamidine 2.5 nM; Suramin 27 nM. Methyl methanesulfonate (MMS, Sigma) was used at 0.0003%. To monitor cumulative trypanosome growth, cultures were grown in the presence of drug, with four technical replicates of cell density counted at 6, 9, 24, 48, 72 and 96 h. Cultures were diluted with fresh media containing the appropriate drug, such that cell density never exceeded 2 x 10^6^ cells.ml^-1^. All tagging and RNAi constructs were transfected into 2T1 *T*. *brucei* cells [50]. RNAi was induced with tetracycline (1 μg/ml) for three days prior to initiating EC_50_ determination.

### Microscopy

Cells were grown in the presence of drug at 5 x EC_50_ for 24 h, unless stated otherwise. Cells were then fixed in 2% v/v paraformaldehyde for 30 min at 4°C before being washed three times in PBS. Fixed cells were dried onto slides before staining with antibodies (outlined below). Slides were washed in PBS and mounted in Vectashield containing DAPI (Vector Laboratories); 4, 6-diamidino-2-phenylindole. Scoring of phenotypes was carried out by counting 100 cells per condition, and by two of us, with the data combined. For immunofluorescence analysis (IFA), cells dried onto slides were permeablised in 0.5% Triton X100 / PBS for 20 min and washed three times in PBS before blocking in 50% foetal bovine serum FBS / PBS. Primary antibody (α-γH2A) [35] was diluted 1:250 and applied for 1 h, slides were washed three times in PBS, and secondary antibody (fluorescein-conjugated goat anti-rabbit) was diluted 1:100 and then applied for 1 h. Antibodies were applied in 3% FBS / PBS. The F1β-subunit of the mitochondrial ATP-synthase was similarly detected using a polyclonal rabbit antiserum directed against the *Crithidia fasciculata* ATP synthase (1:500), which cross-reacts with the *T*. *brucei* orthologue [51,52]. All phase and epifluorescence images were captured on an Eclipse E600 microscope (Nikon) using a Coolsnap FX (Photometrics) charged coupled device camera and processed in Metamorph 5.4 (Photometrics).

### MitoTracker assay

MitoTrackerRed (Invitrogen) was added to cultures at 100 nM. Cultures were incubated for 5 min under standard conditions before parasites were harvested by centrifugation at 1000 x g for 10 min before fixing as outlined above.

### LysoTracker assay

LysoTracker (Invitrogen) was added to cultures at 50 nM. Cultures were incubated under standard conditions for 1 h before parasites were harvested by centrifugation at 1000 x g for 10 min before fixing as outlined above.

### TUNEL assay

Cells on slides were fixed, dried and permeablised as described above for IFA. Reaction mix from the ‘In Situ Cell Death Detection Kit (fluorescein)’ (Roche) was applied to cells for 1 h as per the manufacturer’s instructions.

### Flow cytometry

Cells were harvested and washed in ice cold PBS and resuspended in 300 μl PBS before the addition of 700 μl of methanol and stored at 4°C for 12 h. Cells were then harvested at 400 x g for 10 min at 4°C and resuspended in 1 ml PBS. 10 μg/ml RNAse A and 10 μg/ml propidium iodide was added to each sample and incubated at 37°C for 45 min in the dark. Analysis was performed on an LSRII flow cytometer (BD Biosciences), and data analysis was conducted in FlowJo (Tree Star).

### ImageJ analysis

All images of DAPI fluorescence were captured at 40 ms exposure time for consistency and to avoid overexposure. They were analysed using an ImageJ plug-in [31] modified to enable cell cycle analysis from DAPI and phase contrast images. DAPI alone was suitable for the identification of nuclei and kinetoplasts, since kinetoplasts do not generally overlap with nuclear DNA signals in bloodstream form *T*. *brucei*. As in the original ImageJ plug-in, two kinetoplasts are counted only when no longer linked by pixels with signal. A subset of the original macros were retained and modified, as necessary: Measure K/N Signal, Cell Analysis, K/N Count Summary, and Save Analysis. First, the *Measure K/N Signal* tool thresholds images using built-in ImageJ functions, and extracts object area values from DAPI images. Next, the script applies the K-means clustering algorithm [31] to separate kinetoplasts and nuclei into respective clusters, returning values for the maximum kinetoplast area and minimum nucleus area. These values are then passed to the *Cell Analysis* tool which finds objects in phase images, creates binary copies of the phase and DAPI image and identifies kinetoplasts and nuclei, counting how many lie within each cell. K/N *Count Summary* and *Save Analysis* tools function identically to the original macros described in [31]. Macro scripts are available on request.

### Plasmid construction

For tagging native Tb927.8.5950, Tb927.10.12040 and Tb927.10.1910 alleles with 12-myc epitope tags at the *C*-terminus, we used the following primer-pairs, to amplify 893, 1038 and 769 bp fragments, respectively: 5950MF (GATC*AAGCTT*GATCCATGTGTAGTTGAC) and 5950MR (GATC*TCTAGA*GGATACTGGTGAACCATC); 12040MF (GATC*AAGCTT*GGCACACTTTCACCACGAT) and 12040MR (GATC*TCTAGA*CTCAACGGAACCCACATATT); 1910MF (GATC*AAGCTT*GCGTGTATCTAGGCATGGA) and 1910MR (GATC*TCTAGA*AAGGGAAAAAAGTG). These primer-pairs incorporate HindIII and XbaI sites, respectively (italics). The resulting fragments were cloned in the pNATx^12MYC^ construct [53]. The resulting pNAT^5950-12myc^, pNAT^12040-12myc^ and pNAT^1910-12myc^ constructs were linearized by digestion with Bstz171, EcoRV and EcoRV prior to transfection, respectively. For knockdown of Tb927.8.5950 or Tb927.10.12040 using RNA interference, we used the following primer-pairs, respectively: 5950RF2 (GATC*TCTAGAGGATCC*AAACGACCCAAGTTGGAGAG) and 5950RR2 (GATC*GGGCCCGGTACC*GCTTCCAGCGTCCATGTATT); 12040RF2 (GATC*TCTAGAGGATCC*ATTCTTGGTGAGTTGCTGGG) and 12040RR2 (GATC*GGGCCCGGTACC*ACTCTCATCATACACCGCCC). These primer-pairs incorporate XbaI / BamHI and ApaI / KpnI sites, respectively (italics). The resulting fragments were cloned in the pRPa^iSL^ construct [53]. The resulting pRPa^5950-RNAi^ and pRPa^12040-RNAi^ constructs were linearized by digestion with AscI prior to transfection.

### Protein blotting

Total cell extracts were separated on SDS-polyacrylamide gels and subjected to standard western blotting analysis. Duplicate gels were generated and one was stained with Coomassie and the other was used to produce the nitrocellulose blot. Blots were blocked in 5% milk in TBST and washes were performed in TBST (0.05% Tween). Blots were then probed with mouse α-c-Myc primary antibody (1:5000; 9E10, Source Biosciences) or the ATP synthase primary antibody (1:500). Following incubation with the appropriate secondary antibodies (1:2000; Pierce), membrane was washed and visualised using the Amersham enchanced chemiluminescence (ECL) detection system (GE Healthcare Life Sciences) according to the manufacturer instructions.
